# MiR-34a-3p suppresses pulmonary vascular proliferation in acute pulmonary embolism rat by targeting DUSP1

**DOI:** 10.1042/BSR20210116

**Published:** 2021-12-23

**Authors:** Yang Li, Jinyan Shao, Jianfeng Song, Shuili Yu, Jiqin Wang, Keyu Sun

**Affiliations:** Department of Emergency, Minhang Hospital, Fudan University, Shanghai, China

**Keywords:** acute pulmonary embolism, DUSP1, miR-34a-3p, proliferation, pulmonary artery smooth muscle cells

## Abstract

Background: Acute pulmonary embolism (APE) is a prevalent reason of cardiovascular morbidity and mortality. Recent studies have underscored the positive effects of microRNAs (miRNAs) on many diseases. The present study aimed to identify the critical miRNA with differential expressions and explore its role in APE.

Methods: The critical miRNA with its target gene was screened by bioinformatics analysis. Their binding relationship was analyzed by TargetScan, Dual-luciferase reporter and RNA pull-down assays. A rat model of APE was established by self-blood coagulum. Human pulmonary artery smooth muscle cells (PASMCs) were exposed to platelet-derived growth factor (PDGF-BB) for excessive proliferation, and transfected with miR-34a-3p mimic. Mean pulmonary arterial pressure (mPAP) of rat was measured, and the pulmonary tissues were used for the pathological observation by Hematoxylin–Eosin (H&E) staining. Cell viability and proliferation were detected by Cell Counting Kit-8 (CCK-8) and EdU assays. The expressions of miR-34a-3p with its target genes (including dual-specificity phosphatase-1 (DUSP1)), neuron-derived orphan receptor-1 (NOR-1) and proliferating cell nuclear antigen (PCNA) were determined by quantitative reverse transcription polymerase chain reaction (RT-qPCR) or/and Western blot.

Results: MiR-34a-3p expression was down-regulated in APE patients, which attenuated the increment of mPAP and thickening of the pulmonary arterial walls in APE rats, accompanied with regulation of NOR-1 and PCNA levels. MiR-34a-3p suppressed DUSP1 expression by directly binding to its 3′-untranslated region (UTR), and attenuated cell viability, proliferation, and the expressions of NOR-1 and PCNA in PDGF-BB-induced PASMCs by inhibiting DUSP1 expression.

Conclusion: Up-regulated miR-34a-3p negatively regulates DUSP1 expression to inhibit PASMC proliferation, which, thus, may act on APE treatment by negatively regulating pulmonary vascular proliferation.

## Introduction

In clinical trial, acute pulmonary embolism (APE) is a prevalent cardiovascular disease, which belongs to the most severe venous thromboembolisms [1]. Despite advances in diagnostic paradigm and therapeutic strategies of APE, mortality remains high [2]. It has been reported that pulmonary artery smooth muscle cells (PASMCs) migrate to pulmonary artery intima through excessive proliferation and migration after APE, which leads to pulmonary vasculature reconstruction, thereby enhancing pulmonary vascular resistance [3,4]. Since abnormal proliferation of PASMCs is a common feature of pathological pulmonary vasculature [5], blocking the excessive proliferation of PASMCs may be a potential therapeutic option following APE.

MicroRNAs (miRNAs/miRs) are a class of small noncoding RNAs with over 22 nucleotides that modulate target gene expression at the post-transcriptional level by targeting their 3′ untranslated regions (UTRs). MiRNAs occupy a vital position in different cellular properties, such as cell growth, differentiation and apoptosis [6]. Accumulative reports have demonstrated that miRNAs could be perceived as potent circulating biomarkers by virtue of their high diagnostic and prognostic power in cardiovascular diseases [7–10]. Additionally, some miRNAs have been considered as biomarkers for APE and chronic thromboembolic pulmonary hypertension [11]. Recent reports have proposed the role of miRNAs as biomarkers in APE, as evidenced by expression levels of many miRNAs including miR-23a [12], miR-221 [13], miR-27a/b [14], miR-1233 [15], miRNA-134 [16] and miR-28-3p [17] were obviously elevated in plasma of patients with APE as compared with healthy people. Furthermore, increasing evidence unveiled that many miRNAs could regulate the proliferation of human PASMCs, such as Let-7d [18], miR-23a [19], miR-17 [20], miR-19a [21] and miR-18a-5p [22]. The reports above demonstrated that miRNA may exert an important effect on the proliferation of human PASMCs in APE patients. However, the roles and the underlying mechanisms of specific miRNAs in APE remain to be elucidated. To further reveal the potential mechanisms of APE progression, additional research exploring the functions of miRNAs on APE is necessary.

Therefore, in the present study, we identified a critical miRNA by bioinformatics analysis, further revealing its role in the thickening of rat pulmonary arterial walls in an APE model *in vivo* and in excessive proliferation of human platelet-derived growth factor (PDGF-BB)-induced PASMCs *in vitro*, as well as determining the underlying molecular mechanisms.

## Materials and methods

### Study subjects

A total of 42 patients diagnosed with APE and without cardiopulmonary diseases between 2018 and 2019 at Minhang Hospital, Fudan University were selected for the present study. APE in patients was diagnosed by computed tomographic pulmonary angiography (CTPA). Plasma from APE patients (*n*=42) and healthy people (*n*=42) was harvested.

### Animal grouping

A total of 50 male Sprague–Dawley rats (7 weeks old, weighing 250 ± 20 g) were obtained from the Vital River Laboratory Animal Technology Co., Ltd. (Beijing, China). The rats that reared in Minhang Hospital, Fudan University with *ad libitum* access to water and food were assigned to the sham operation group, APE group, APE+scramble group, APE+miR-34a-3p-agomir group and APE+miR-34a-3p-antagomir group, with ten rats in each group.

### Establishment of APE rat model and treatment

The APE rat model was established through the autologous blood clot method, as previously described [3]. In short, after coagulation of blood that was collected from the tail veins of the rats, blood clots were incubated in 70°C water bath for 10 min and subsequently diced into 1.1 mm × 2 mm size. Then, 25 autologous blood clots with 2 ml were injected. AgomiR-34a-3p (5′-AAUCAGCAAGUAUACUGCCCUA-3′), scramble (5′-UUCUCCGAACGUGUCACGU-3′) and antagomiR-34a-3p (5′-UAGGGCAGUAUACUUGCUGAUU-3′) were purchased from Ribobio Co., Guangzhou, China and the mixture (100 μg agomiR-34a-3p or scramble or antagomiR-34a-3p with 50 μl transfection reagent (18668-11-1; Engreen, Beijing, China)) was prepared as previously described [23]. The rats were respectively intravenously injected with agomiR-34a-3p, scramble or antagomiR-34a-3p for 3 consecutive days through tail vein, 15 min after model establishment, as previously described [23,24]. Sham and APE groups were exposed to tail vein injection of the same amount of normal saline.

### Measurement of mean pulmonary arterial pressure

By day 7, the rats were anesthetized with 1 g/kg body weight of urethane (U820333, Macklin Shanghai, China, intraperitoneally [i.p.]). The right external jugular vein of rats fixed in a supine position was isolated, a small incision was cut, and the heparin-filled microcatheter was inserted through the right heart to the pulmonary artery. The pressure sensor was connected to the other end of the heparin-filled microcatheter to record the rat mean pulmonary arterial pressure (mPAP) with a Gould-3400s/DASA4600 (American) physiological recorder. At the end of the experiment, the rats were anesthetized as mentioned above and their necks were dislocated for euthanasia. The bilateral pulmonary tissues were stored at −70°C following dissection, and used for the pathological observation of pulmonary vascular morphology by Hematoxylin–Eosin (H&E) staining and for the detection of miR-34a-3p, neuron-derived orphan receptor-1 (NOR-1) and proliferating cell nuclear antigen (PCNA) expressions by quantitative reverse transcription polymerase chain reaction (RT-qPCR).

### Cell culture and treatment

Human PASMCs (CC-2581) were purchased from Lonza (Walkersville, MD, U.S.A.) and cultured in SmGM-2TM smooth muscle growth medium-2 (CC-3182, Lonza) with 5% fetal bovine serum (FBS, 11011-8611, Sijiqing Co., Ltd, Hangzhou, China) and 1% antibiotics (P1400, Solarbio) in a humid incubator at 37°C with 5% CO_2_. For proliferation induction, cells were exposed to 20 ng/ml PDGF-BB (220-BB, R&D Systems, Minneapolis, MN, U.S.A.) for 24 h, as previously mentioned [25].

### Transfection

MiR-34a-3p mimic (M; miR10004557-1-5) and mimic control (MC; miR1N0000001-1-5) were obtained from RiboBio (Ribobio Co., Guangzhou, China). Overexpressed dual-specificity phosphatase-1 (DUSP1) plasmid was constructed by pcDNA3.1 vector (V79520, Thermo Fisher Scientific, Rockford, IL, U.S.A.), with the empty pcDNA3.1 vector serving as a negative control (NC). PASMCs were transfected with miR-34a-3p mimic or overexpressed DUSP1 plasmid, and their corresponding negative controls (MC, NC). A total of 1 × 10^6^ PASMCs were subjected to transfection with 50 nM vectors or 30 nM plasmids using 1 ml transfection kit (C10511-05, RiboBio) at 37°C for 6 h, based on the manufacturer’s instructions. After that, the transfected medium was replaced with a complete culture medium, and the PASMCs were further incubated for 24–48 h.

### Bioinformatics analysis

The dataset (GSE24149: MicroRNAs as potential biomarkers for APE) from the Gene Expression Omnibus (GEO) (https://www.ncbi.nlm.nih.gov/geo/) was analyzed using GEO2R online (available at https://www.ncbi.nlm.nih.gov/geo/geo2r/) on two platforms (GPL10922 and GPL10923) to compare differential miRNAs between ten APE patients and healthy controls in plasma.

Prediction of miR-34a-3p target genes was conducted through TargetScan (http://www.targetscan.org), miRDB (http://mirdb.org/miRDB/), miRWalk (www.umm.uni-heidelberg.de/apps/zmf/mirwalk) and RNAcentral websites (https://rnacentral.org/). The binding sites between miR-34a-3p and DUSP1 were predicted by TargetScan.

### H&E staining

H&E staining was performed with H&E Staining Kit (G1120, Solarbio). The paraffin-embedded pulmonary artery sections were dewaxed twice by xylene I at room temperature for 5 min and rehydrated with an ethanol gradient series. The dewaxed sections were soaked in Hematoxylin solution for 20 min, washed with Hematoxylin differentiation solution for 30 s to terminate the staining, and soaked in tap water for 15 min, followed by counterstaining with Eosin solution for 2 min, rinsing and then soaking in tap water for 5 min. The sections were dehydrated with ethanol gradient series (95% ethanol (2 min), 95% ethanol (2 s), 100% ethanol (2 s), 100% ethanol (2 s)), then rendered transparent with xylene solution of carbolic acid for 1 min, followed by soaked twice in xylene (1 min each time), and sealed with neutral gum. The pathological observation was performed by a light microscope (Olympus, BXFM).

### RT-qPCR

The harvested pulmonary tissues were mixed with miRNeasy mini kit (217004, Qiagen, Valencia, CA, U.S.A.) and homogenized with a tissue homogenate machine. The total RNAs and miRNAs from plasma, tissue-grinding solution, or PASMCs were obtained with the use of miRNeasy mini kit. TaqMan™ MicroRNA Reverse Transcription Kit (4366596; Applied Biosystems, Foster City, CA, U.S.A.) was used to synthesize miRNA-specific complementary DNA (cDNA). With respect to mRNA, the isolated RNA was reverse-transcribed into cDNA using PrimeScript™ RT Master Mix (RR036A, TaKaRa, Dalian, Liaoning, China). Quantitative PCR was conducted with SYBR Premix ExTaq II Kit (RR820A, TaKaRa) in ABI7500 quantitative PCR instrument (Applied Biosystems, Oyster Bay, NY, U.S.A.). The amplification reaction was conducted at 95°C for one cycle for 30 s, followed by 40 cycles at 95°C for 3 s, 60°C for 30 s and one cycle at 72°C for 5 min. Expression levels of genes were determined by the relative quantitative method (2^−DD*C*_t_^ method [26]), with GAPDH or U6 used as the internal reference. The primer sequences of target genes are listed in Table 1.

**Table 1:** Specific primer sequences for RT-qPCR

Gene	Primer sequence	Species
*miR-34a-3p*	5′-CCCTGTCGTATCCAGTGCAA-3′	Human
	5′-GTCGTATCCAGTGCGTGTCG-3′	
*U6*	5′-CTCGCTTCGGCAGCACA-3′	Human
	5′-AACGCTTCACGAATTTGCGT-3′	
*NOR-1*	5′-TGCGTCCAAGCCCAATATAGC-3′	Human
	5′-GGTGTATTCCGAGCTGTATGTCT-3′	
*PCNA*	5′-CCTGCTGGGATATTAGCTCCA-3′	Human
	5′-CAGCGGTAGGTGTCGAAGC-3′	
*DUSP1*	5′-AGTACCCCACTCTACGATCAGG-3′	Human
	5′-GAAGCGTGATACGCACTGC-3′	
*ATXN7L3B*	5′-GTGTGGCTACTTCTACCTGGA-3′	Human
	5′-GGCACGCTCCTTTGTCTTC-3′	
*OLA1*	5′-TTGCAGCACTCCAACTAGAATAC-3′	Human
	5′-TCGGTTGTTGAGGTGTGTTAAAT-3′	
*SLC22A5*	5′-TCCACCATTGTGACCGAGTG-3′	Human
	5′-ACCCACGAAGAACAAGGAGATT-3′	
*TMEM107*	5′-CTCCATTGGGGCTCACTGTAG-3′	Human
	5′-ACGGTGACGAATAAAGCCATTT-3′	
*GAPDH*	5′-CCACTCCTCCACCTTTGAC-3′	Human
	5′-ACCCTGTTGCTGTAGCCA-3′	
*miR-34a-3p*	5′-ACTGCCCTAGTCGTATCCAGT-3′	Rat
	5′-GTATCCAGTGCGTGTCGTGG-3′	
*U6*	5′-CTCGCTTCGGCAGCACA-3′	Rat
	5′-AACGCTTCACGAATTTGCGT-3′	
*NOR-1*	5′-CGATGTCAGTACTGCAGGTTTCAG-3′	Rat
	5′-TCTGTACGCACAACTTCCTTCAC-3′	Rat
*PCNA*	5′-GAGTACAGCTGCGTAGTAAA-3′	Rat
	5′-ACTGGCTCATTCATCTCTAT-3′	
*GAPDH*	5′-CAACTCCCTCAAGATTGTCAGCAA-3′	Rat
	5′-GGCATGGACTGTGGTCATGA-3′	

### Dual-luciferase reporter assay

Sequences of DUSP1 (Wildtype: 5′-AACAGTTGTATGTTTGCTGATTA-3′, Mutant-Type: 5′-AACAGTTGTATGTTTGATGGCTA-3′) were located into pmirGLO vectors (E1330, Promega, Madison, WI, U.S.A.). Then, PASMCs were co-transfected with miR-34a-3p mimic or mimic control and pmirGLO vectors containing DUSP1 sequences by Lipofectamine 3000 reagent. After 48 h transfection, the fluorescence intensity of each group was detected by dual-luciferase reporter assay system (E1980, Promega).

### RNA pull-down assay using streptavidin magnetic beads

RNA pull-down assay was conducted as per the instructions of Pierce™ RNA Pull-Down Kit (20164, Thermo Scientific). DUSP1 and DUSP1 antisense (served as negative control) were ligated into pcNDA 3.1 vector and subsequently transcribed *in vitro* using T7 RNA polymerase (p2075, Promega). These RNAs were labeled by biotin using Pierce™ RNA 3′ End Desthiobiotinylation, and the mixture was treated with prewashed Pierce™ Streptavidin Magnetic Beads. Then, these beads were incubated with cell extracts at 4°C overnight. After washing non-specific binding, the hybridized RNAs were extracted using TRIzol and detected by RT-qPCR.

### Western blot

Total protein was extracted by RIPA Buffer (89900, Thermo Fisher, U.S.A.) and quantified with BCA kits (A53227, Thermo Fisher, U.S.A.). The harvested protein samples with the protein-loading buffer (P0015, Beyotime) were boiled for 5 min, and loaded into 10% SDS/PAGE gel for electrophoresis separation with ColorMixed Protein Marker (11–180 kDa, PR1910, Solarbio). Then, the protein was transferred on to PVDF membrane (YA1701, Solarbio) and blocked with 5% bovine serum albumin (BSA) (SW3015, Solarbio) at 37°C for 2 h. The membrane was incubated firstly with the primary antibodies (DUSP1 (#48625, Rabbit, 1:1000, CST, U.S.A.), NOR-1 (ab155535, Rabbit, 1:3000, Abcam, Cambridge, MA, U.S.A.), PCNA (ab29, Mouse, 1:1000, Abcam), GAPDH (Mouse, ab8245, 1:10000, Abcam)) at 4°C overnight, and subsequently with the secondary antibody (Goat Anti-Rabbit IgG H&L (HRP) (ab205718, 1:20000, Abcam) or Goat Anti-Mouse IgG H&L (HRP) (ab205719, 1:20000, Abcam)) for 1 h. Then, the membrane was developed by the BeyoECL Moon (P0018FS, Beyotime) in the dark. The protein signal was detected using Bio-Rad gel imaging system (ChemiDocXRS + Imaging System), with GAPDH serving as the internal reference. The original Western blot images were shown in Supplementary Figures S1 and S2.

### Cell counting kit-8 assay

To understand the effects of miR-34a-3p and DUSP1 on PASMC viability, the effects of miR-34a-3p and DUSP1 on 24 h proliferation were measured with CCK-8 assay kit (CK04, Solarbio) in accordance with manufacturer’s instructions. After transfection, 1 × 10^4^ PASMCs were planted into 96-well plates and grown to ∼80% confluence. The serum was then starved for 24 h before experiment. PASMCs were inducted by 20 ng/ml PDGF-BB for 24 h, followed by 10 μl Cell Counting Kit-8 (CCK-8) solution for 4 h. Cell proliferation was detected through monitoring the absorbance at 450 nm under a microplate reader (Molecular Devices, Sunnyvale, CA, U.S.A.).

### EdU assay

PASMCS proliferation was examined using EdU cell proliferation assay kit (CA1170, Solarbio) in line with manufacturer’s instructions. Cells in each well were incubated with 100 ml of 50 μM EdU medium for 2 h and fixed with 50 μl of 4% paraformaldehyde for half an hour. Subsequently, PASMCs in each well were added with 50 ml of 2 mg/ml glycine (G8200, Solarbio), and incubation was performed in a decolorized shaker for 5 min. Next, the PASMCs were exposed to 100 ml of 0.5% Triton X-100 (P1080, Solarbio) for 10 min and then added with 100 ml of 1× Apollo staining reaction solution for 30 min in the dark. After that, the PASMCs were washed with 100 ml of 0.5% Triton X-100 and added with 100 ml methanol at a time. The PASMCs were subsequently incubated with 100 ml of 1× Hoechst 33342 reaction solution (prepared from Reagent F) for 30 min in the dark, and immediately observed with a fluorescent microscope (Olympus, BXFM).

### Data analysis

The experiment was independently carried out in triplicate. Data analysis was conducted using GraphPad Prism software 8.0, and measurement data were expressed as mean ± standard deviation (SD). The independent-sample *t* test was used for comparing two groups, while one-way analysis of variance (ANOVA) was for multiple groups followed by Tukey’s multiple comparison test for pairwise comparison. *P*<0.05 was considered as statistical significance.

## Results

### MiR-34a-3p expression was down-regulated in APE patients

**Figure 1:**
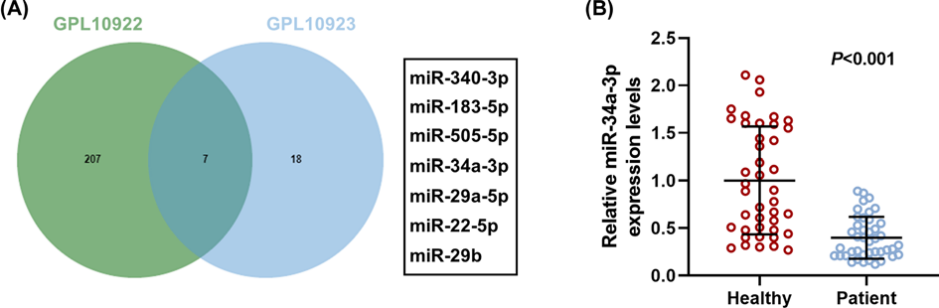
MiR-34a-3p expression was down-regulated in APE patients (**A**) Seven miRNAs were screened from two platforms (GPL10922 and GPL10923) through comparing differential miRNAs between ten APE patients and ten healthy controls in plasma. (**B**) RT-qPCR indicated miR-34a-3p level in 42 APE patients and 42 healthy controls in plasma. U6 was served as the internal control.

From Figure 1A, TaqMan miRNA assay performed by GPL10922 and GPL10923 platforms using GEO2R discovered seven overlapping differential miRNAs between ten pairs of APE patients and healthy controls in plasma. Among them, expressions of six miRNAs (miR-340-3p, miR-183-5p, miR-505-5p, miR-29a-5p, miR-22-5p and miR-29b) were notably down-regulated in APE patients from GPL10922 platform but were markedly elevated from GPL10923 platform, as compared with healthy controls. However, differing from other miRNAs, miR-34a-3p expression was dramatically down-regulated in APE patients from the two platforms as compared with healthy controls, while the same trend was confirmed by detection of RT-qPCR in plasma of APE patients (Figure 1B, *P*<0.001).

### MiR-34a-3p attenuated the increase in mPAP and the thickening of the pulmonary arterial walls in APE rats

**Figure 2:**
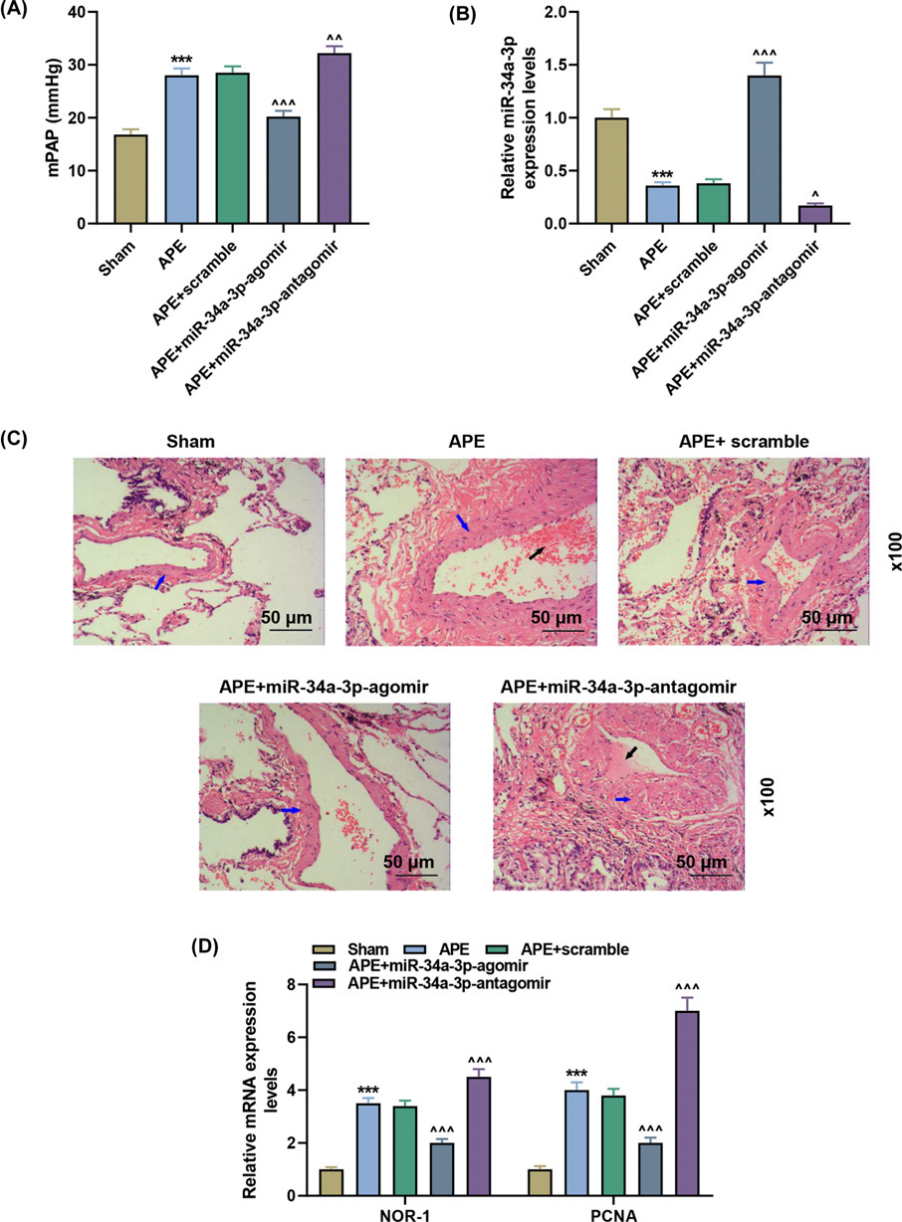
MiR-34a-3p attenuated the increase in mPAP and the thickening of the pulmonary arterial walls in APE rats (**A**) mPAP level was measured in rats. (**B**) RT-qPCR indicated miR-34a-3p level in pulmonary tissues of rats. (**C**) H&E staining was used for pathological observation of pulmonary tissues. Blue arrows indicate the pulmonary artery wall, while black arrows indicate the thrombus. (**D**) RT-qPCR indicated NOR-1 and PCNA levels in pulmonary tissues of rats. ***P<0.001 vs. Sham; ^∧^*P*<0.05, ^∧∧^*P*<0.01, ^∧∧∧^*P*<0.001 vs. APE+scramble. Scramble: negative control. Data are presented as mean ± SD (*n*=10 in each group).

Increased mPAP level and decreased miR-34a-3p level were observed in APE group as compared with sham group (Figure 2A, *P*<0.001; Figure 2B, *P*<0.001), while the two tendencies were partially counteracted by miR-34a-3p agomir (Figure 2A, *P*<0.001; Figure 2B, *P*<0.001), but further enhanced by miR-34a-3p antagomir (Figure 2A, *P*<0.01; Figure 2B, *P*<0.05). From Figure 2C, the H&E staining results demonstrated that 1 week after the embolism, the thrombus was partially dissolved and the pulmonary artery walls were thickened in APE and APE+scramble groups, which were also partially reversed by miR-34a-3p agomir, but further promoted by miR-34a-3p antagomir. In addition, the levels of NOR-1 and PCNA were increased in pulmonary arterial walls of APE rats (Figure 2D, *P*<0.001), experiencing similar aforementioned effects of miR-34a-3p agomir (Figure 2D, *P*<0.001) and miR-34a-3p antagomir (Figure 2D, *P*<0.001).

### MiR-34a-3p suppressed DUSP1 expression by directly binding to its 3′-UTR

**Figure 3:**
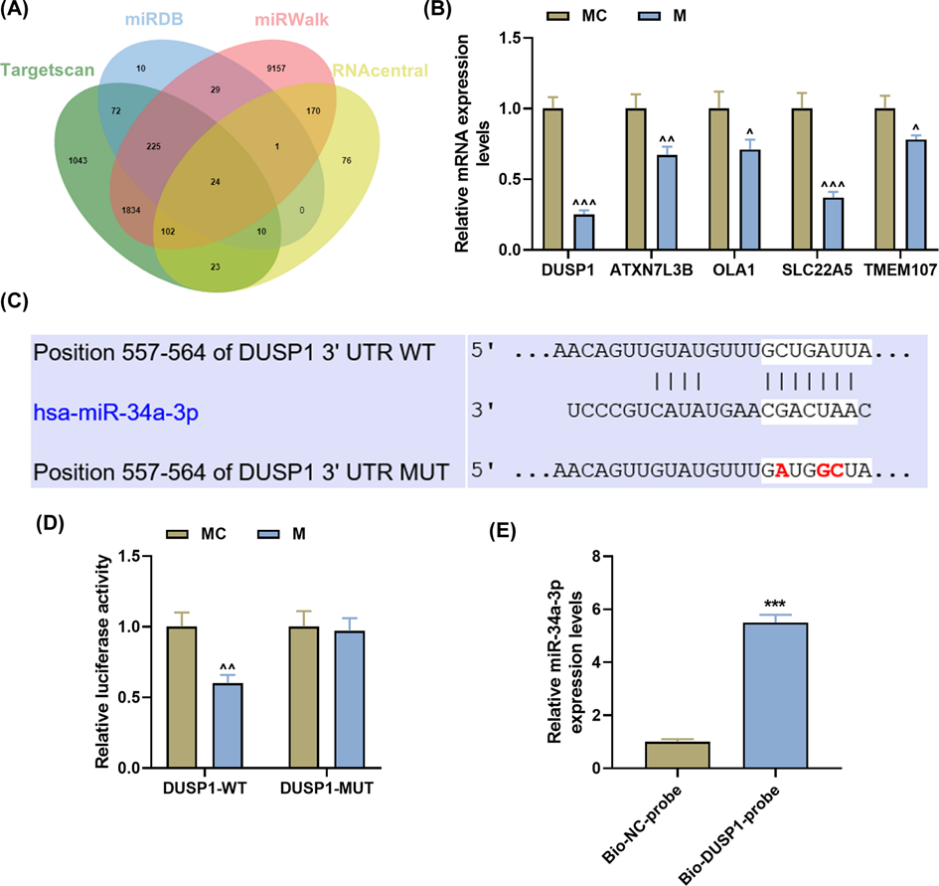
MiR-34a-3p suppressed DUSP1 expression by directly binding to its 3′-UTR (**A**) Prediction of miR-34a-3p target genes was conducted through TargetScan, miRDB, miRWalk and RNAcentral websites. (**B**) RT-qPCR indicated the levels of DUSP1, ATXN7L3B, OLA1, SLC22A5 and TMEM107 in PASMCs. (**C–E**) The binding relationship between miR-34a-3p and DUSP1 was predicted by TargetScan, and verified by dual-luciferase reporter (**D**) and RNA pull-down assays (**E**). PASMCs, pulmonary artery smooth muscle cells; 3′-UTR, 3′-untranslated region; RT-qPCR, quantitative reverse transcription polymerase chain reaction; M, miR-34a-3p mimic; Bio-NC-probe, biotin-labeled RNA. ^∧^*P*<0.05, ^∧∧^*P*<0.01, ^∧∧∧^*P*<0.001 vs. MC; ***P<0.001 vs. Bio-NC-probe. Data are presented as mean ± SD (with *n*=3 in each group).

As depicted in Figure 3A, 24 overlapping downstream genes could be regulated by miR-34a-3p, which were predicted by TargetScan, miRDB, miRWalk and RNAcentral. The top five genes (*DUSP1*, *ATXN7L3B*, *OLA1*, *SLC22A5*, *TMEM107*) with the highest binding site score were selected for further study, among which DUSP1 was the most significant differential gene in miR-34a-3p mimic group (three-fold decrease, Figure 3B, *P*<0.001). It was found that the binding site between miR-34a-3p and DUSP1 was predicted by TargetScan (Figure 3C), with their binding relationship being further verified by dual-luciferase reporter (Figure 3D, *P*<0.001) and RNA pull-down results (Figure 3E, *P*<0.01).

### MiR-34a-3p attenuated the increase in DUSP1 expression in PDGF-BB-induced PASMCs

**Figure 4:**
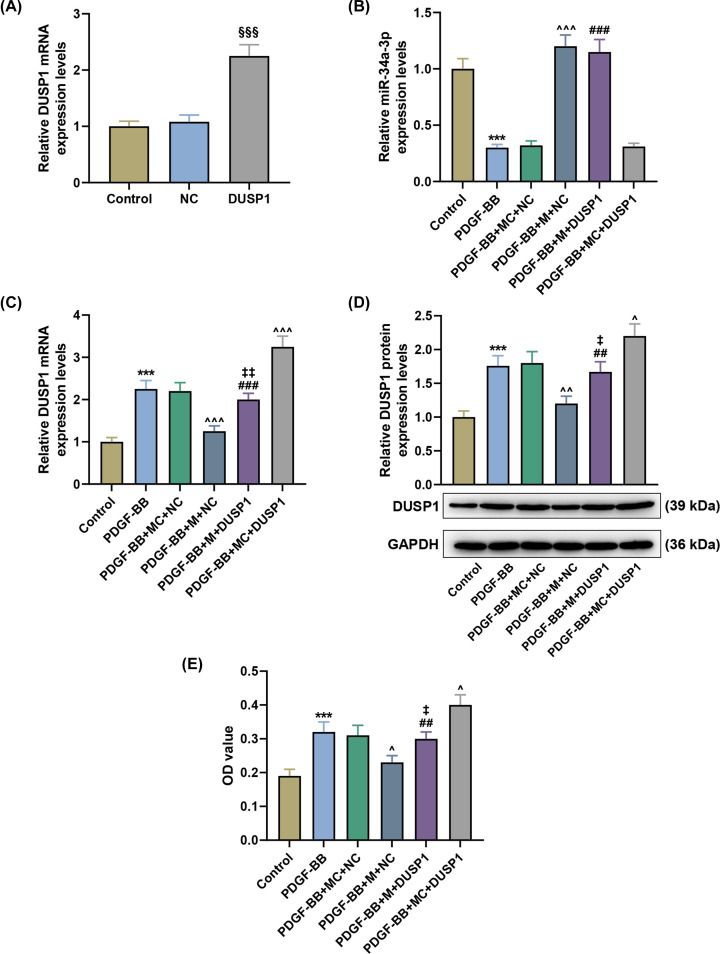
MiR-34a-3p attenuated the increase in DUSP1 expression in PDGF-BB-induced PASMCs (**A**) RT-qPCR indicated the level of DUSP1 in PASMCs following transfection with DUSP1 plasmid. (**B**) RT-qPCR indicated the level of miR-34a-3p in PASMCs after PDGF-BB induction and nucleic acid transfection. (**C,D**) Western blot indicated the level of DUSP1 in PASMCs after PDGF-BB induction and nucleic acid transfection in PASMCs. (**E**) CCK-8 indicated cell viability of PASMCs after PDGF-BB induction and nucleic acid transfection. U6 was used as the internal control for miR-34a-3p. GAPDH was used as the internal control for DUSP1. Control, without any treatment; NC, negative control for DUSP1 plasmid; M, miR-34s-3p mimic; M, miR-34a-3p mimic. ***P<0.001 vs. Control; ^∧^*P*<0.05, ^∧∧^*P*<0.01, ^∧∧∧^*P*<0.001 vs. PDGF-BB+MC+NC; ^##^*P*<0.01, ^###^*P*<0.001 vs. PDGF-BB+MC+DUSP1; ^‡^*P*<0.05, ^‡‡^*P*<0.01 vs. PDGF-BB+M+NC; ^§§§^*P*<0.001 vs. NC. Data are presented as mean ± SD (*n*=3 in each group).

It can be noted from Figure 4A that DUSP1 plasmid successfully promoted DUSP1 mRNA expression in PASMCs (*P*<0.001). PDGF-BB induced down-regulation of miR-34a-3p expression and up-regulation of DUSP1 mRNA (Figure 4B, *P*<0.001; Figure 4C, *P*<0.001). However, miR-34a-3p mimic exerted the opposite effects to PDGF-BB on expressions of miR-34a-3p and DUSP1 mRNA (Figure 4B, *P*<0.001; Figure 4C, *P*<0.001), which was partially offset by DUSP1 plasmid (Figure 4C, *P*<0.001). Moreover, DUSP1 plasmid did not affect the expression of miR-34a-3p (Figure 4B), but remarkably up-regulated that of DUSP1 in PDGF-BB-induced PASMCs (Figure 4C,D, *P*<0.05).

### MiR-34a-3p attenuated the increase in cell viability, proliferation and the protein expressions of NOR-1 and PCNA in PDGF-BB-induced PASMCs by inhibiting DUSP1 expression

**Figure 5:**
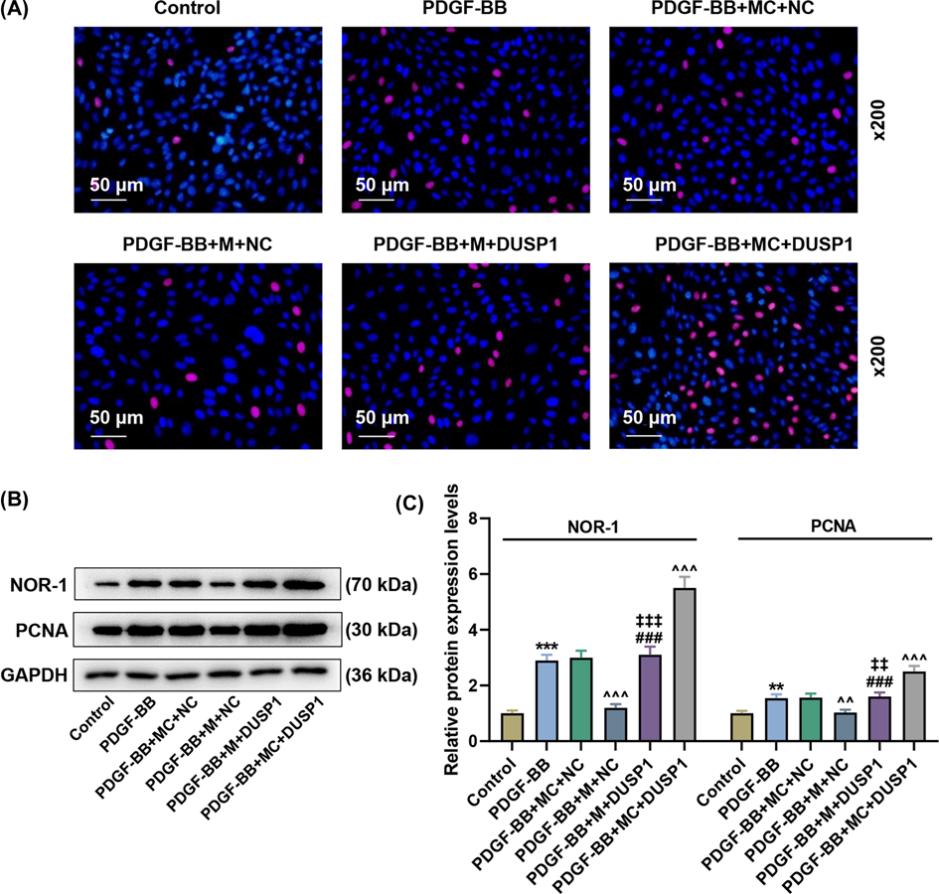
MiR-34a-3p attenuated the increase in cell viability, proliferation and the protein expressions of NOR-1 and PCNA in PDGF-BB-induced PASMCs by inhibiting DUSP1 expression (**A**) EdU assay indicated cell proliferation of PASMCs. (**B**,**C**) Western blot indicated the levels of NOR-1 and PCNA in PASMCs. GAPDH was used as the internal control. M, miR-34s-3p mimic; NC, negative control for DUSP1 plasmid. **P<0.01, ***P<0.001 vs. Control; ^∧∧^*P*<0.01, ^∧∧∧^*P*<0.001 vs. PDGF-BB+MC+NC; ^###^*P*<0.001 vs. PDGF-BB+MC+DUSP1; ^‡‡^*P*<0.01, ^‡‡‡^*P*<0.001 vs. PDGF-BB+M+NC. Data are presented as mean ± SD, with *n*=3 in each group.

PDGF-BB promoted cell viability, cell proliferation and the protein expressions of NOR-1 and PCNA in PASMCs (Figure 4E, *P*<0.001; Figure 5A–C, *P<*0.01), which was partially reversed by miR-34a-3 mimic (Figure 4E, *P<*0.05; Figure 5A–C, *P<*0.01) but further enhanced by DUSP1 overexpression (Figure 4E, *P<*0.05; Figure 5A–C, *P<*0.001). Moreover, DUSP1 overexpression counteracted the effect of miR-34a-3 mimic on cell viability, cell proliferation and the protein expressions of NOR-1 and PCNA in PDGF-BB-induced PASMCs (Figure 4E, *P<*0.05; Figure 5A–C, *P<*0.05).

## Discussion

In our work, miR-34a-3 expression was down-regulated in APE patients, APE rat model and PDGF-BB-induced PASMCs. MiR-34a-3 suppressed pulmonary vascular proliferation in APE rat model and the excessive proliferation in the PDGF-BB-induced PASMCs by targeting DUSP1.

APE, despite being common, often remains elusive in diagnosis, so a high degree of clinical suspicion needs to be maintained when treating a patient with cardiopulmonary symptoms [27]. Diagnostic algorithms and techniques have remained relatively unchanged over the past decade, among which CTPA is the principal tool, however, it is inappropriate for all cases of suspected APE [27]. Besides, most clinical presentations are nonspecific, which may lead to frequent misdiagnosis [28,29]. Multiple studies have described remarkable stability of extracellular miRNAs, in spite of the hostile extracellular environment [30]. On account of the remarkable stability in body fluids, miRNAs may serve as novel diagnostic biomarkers of APE. Increasing evidence has verified miR-34a-3p as a reliable biomarker in the diagnosis of different diseases [31–33]. The present study showed that seven down-regulated miRNAs were screened in APE patients and compared with healthy controls by bioinformatics analysis. Differing from other miRNAs, miR-34a-3p expression was down-regulated in APE patients from GPL10922 and GPL10923 platforms and in plasma of APE patients as compared with healthy controls. A similar report also demonstrated that circulatory miR-34a-3p expression is decreased in both patients with pulmonary arterial hypertension (PAH) and preclinical models of PAH [34]. Therefore, miR-34a-3p may serve as a potential biomarker and therapeutic target in APE.

Consistent with above results in our study, miR-34a-3p expression was also down-regulated in APE rats, which further enhanced the increase in mPAP and the thickening of the pulmonary arterial walls in APE rats, whereas up-regulated miR-34a-3p showed opposite results. NOR-1 activity persisted at a relatively low expression in healthy vascular endothelial cells and elevated under the influence of external stimuli [35,36]. As a modulator of inflammation, growth factors, lipoproteins and thrombin, NOR-1 regulates the proliferation of vascular cells [37–40], which is also reported to control the proliferation of mouse PASMCs involved with miR-106b-5p [24]. In addition, as an auxiliary protein for DNA polymerase δ, PCNA is believed to be critical to regulating cell proliferation [41,42]. Hence, the detection of NOR-1 and PCNA expression levels in pulmonary arterial tissues allows the evaluation of pulmonary arterial proliferation. Thus, the data suggested that up-regulated miR-34a-3p attenuated pulmonary arterial proliferation in APE rats, with manifestations of increased pulmonary arterial walls thickening as well as NOR-1 and PCNA levels. Hence, up-regulated miR-34a-3p attenuated pulmonary arterial vascular obstruction in APE rats through the above mechanisms. The data also indicated that miR-34a-3p occupies a critical position in pulmonary arterial vascular obstruction of APE, which may act as a promising target for the treatment of APE.

Vascular contraction, a major function of vasoconstriction, can regulate vascular tension and diameter, thereby controlling blood pressure and blood flow distribution [43]. PDGF-BB, a potent mitogen for vascular smooth muscle cells (VSMCs), plays a pivotal role in inducing the phenotypic switching of VSMCs from contractile to proliferative state [43]. We therefore constructed excessive proliferation of human PASMCs by means of PDGF-BB induction. MiR-34a-3p promoted proliferation in diverse types of cells, such as meningioma cells [44] and rheumatoid arthritis fibroblast-like synoviocytes [45], but exerted the opposite effect on cervical cancer cells [46]. Furthermore, DUSP1 also made profound impacts upon proliferation in many types of cells, including keratinocytes [47], gallbladder cancer cells [48], high glucose-induced cardiac fibroblasts [49] and small cell carcinoma of the prostate PC-3 cells [50]. The current study revealed that the expression of miR-34a-3p was down-regulated in PDGF-BB-mediated PASMCs, while DUSP1 mRNA and protein expressions were up-regulated. Besides, the present data proved that miR-34a-3p suppressed DUSP1 expression by binding to its 3′-UTR in PDGF-BB-induced PASMCs, which may attenuate the increase in cell viability, proliferation, and the protein expressions of NOR-1 and PCNA in PDGF-BB-induced PASMCs, thus confirming the role of miR-34a-3p as a potential biomarker and therapeutic target for APE.

## Conclusion

Our study demonstrates that the up-regulation of miR-34a-3p negatively regulates DUSP1 expression to inhibit PASMCs proliferation, which, therefore, may be a potential target for the treatment of APE by negatively regulating pulmonary vascular proliferation.

## Supplementary Material

Supplementary Figures S1-S2

## Data Availability

The analyzed datasets generated during the study are available from the corresponding authors on reasonable request.

## Abbreviations

APE: acute pulmonary embolism
CCK-8: cell counting kit-8
cDNA: complementary DNA
CTPA: computed tomographic pulmonary angiography
DUSP1: dual-specificity phosphatase-1
EdU: 5-Ethynyl-2′-deoxyuridine
HPR: Horseradish peroxidase
H&E: Hematoxylin–Eosin
MC: mimic control
miR/miRNA: microRNA
mPAP: mean pulmonary arterial pressure
NC: negative control
NOR-1: neuron-derived orphan receptor-1
PASMC: pulmonary artery smooth muscle cell
PCNA: proliferating cell nuclear antigen
PDGF-BB: platelet-derived growth factor
RT-qPCR: quantitative reverse transcription polymerase chain reaction
UTR: untranslated region
VSMC: vascular smooth muscle cell
