# Supplementary material for: MiR-34a-3p suppresses pulmonary vascular proliferation in acute pulmonary embolism rat by targeting DUSP1
Source: Biosci Rep. 2021 Dec 23;42(1):BSR20210116. doi: 10.1042/BSR20210116 (PMC8703022; doi:10.1042/BSR20210116)
Supplement: Supplementary Figures S1-S2 [file BSR-2021-0116_supp.pdf]

Supplementary Fig. 1

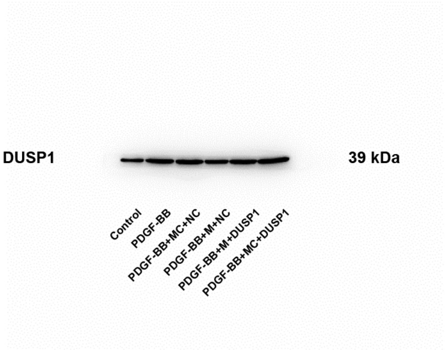

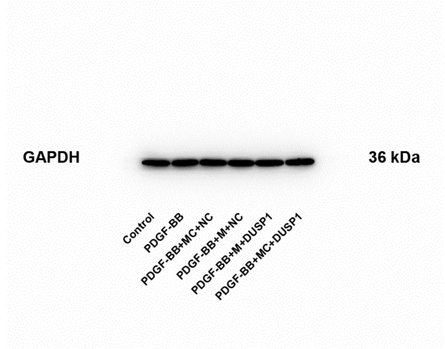

Supplementary figure 1. Representative blots for western blot experiment in Fig. 4D. Representative blots showed DUSP1 expression in PDGF-BB-induced PASMCs. GAPDH was used as a loading control. PASMCs, pulmonary artery smooth muscle cells; PDGF-BB, platelet-derived growth factor; Control, without any treatment; NC, negative control for DUSP1 plasmid; M, miR-34s-3p mimic; M, miR-34a-3p mimic; MC, mimic control.

Supplementary Fig. 2

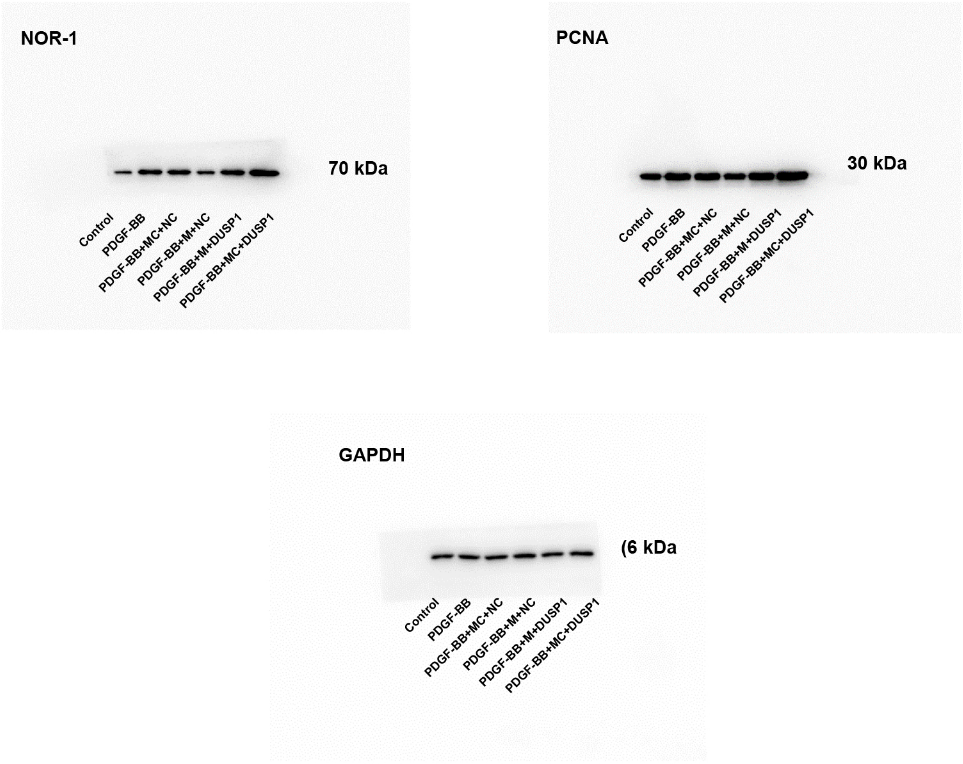

Supplementary figure 2. Representative blots for western blot experiment in Fig. 5B. Representative blots showed NOR-1 and PCNA protein expressions in PDGF-BB-induced PASMCs. GAPDH was used as a loading control. PASMCs, pulmonary artery smooth muscle cells; PDGF-BB, platelet-derived growth factor; Control, without any treatment; NC, negative control for DUSP1 plasmid; M, miR-34s-3p mimic; M, miR-34a-3p mimic; MC, mimic control.
